# Immunogenic Properties of Archaeal Species Found in Bioaerosols

**DOI:** 10.1371/journal.pone.0023326

**Published:** 2011-08-12

**Authors:** Pascale Blais Lecours, Caroline Duchaine, Michel Taillefer, Claudine Tremblay, Marc Veillette, Yvon Cormier, David Marsolais

**Affiliations:** 1 Centre de recherche de l'Institut Universitaire de Cardiologie et de Pneumologie de Québec, Québec, Canada; 2 Département de biochimie, de microbiologie et de bioinformatique, Faculté des sciences et de génie, Université Laval, Québec, Canada; 3 Laboratoires Charles River, Services Précliniques, Montréal, Canada; 4 Départment de médecine, Faculté de médecine, Université Laval, Québec, Canada; Centre de Recherche Public de la Santé (CRP-Santé), Luxembourg

## Abstract

The etiology of bioaerosol-related pulmonary diseases remains poorly understood. Recently, archaea emerged as prominent airborne components of agricultural environments, but the consequences of airway exposure to archaea remain unknown. Since subcomponents of archaea can be immunogenic, we used a murine model to study the pulmonary immune responses to two archaeal species found in agricultural facilities: *Methanobrevibacter smithii* (MBS) and *Methanosphaera stadtmanae* (MSS). Mice were administered intranasally with 6.25, 25 or 100 µg of MBS or MSS, once daily, 3 days a week, for 3 weeks. MSS induced more severe histopathological alterations than MBS with perivascular accumulation of granulocytes, pronounced thickening of the alveolar septa, alveolar macrophages accumulation and increased perivascular mononucleated cell accumulation. Analyses of bronchoalveolar lavage fluids revealed up to 3 times greater leukocyte accumulation with MSS compared to MBS. Instillation of 100 µg of MBS or MSS caused predominant accumulation of monocyte/macrophages (4.5×10^5^ and 4.8×10^5^ cells/ml respectively) followed by CD4^+^ T cells (1.38×10^5^ and 1.94×10^5^ cells/ml respectively), B cells (0.73×10^5^ and 1.28×10^5^ cells/ml respectively), and CD8^+^ T cells (0.20×10^5^ and 0.31×10^5^ cells/ml respectively) in the airways. Both archaeal species induced similar titers of antigen-specific IgGs in plasma. MSS but not MBS caused an accumulation of eosinophils and neutrophils in the lungs, which surprisingly, correlated inversely with the size of the inoculum. Stronger immunogenicity of MSS was confirmed by a 3 fold higher accumulation of myeloid dendritic cells in the airways, compared to MBS. Thus, the dose and species of archaea determine the magnitude and nature of the pulmonary immune response. This is the first report of an immunomodulatory role of archaeal species found in bioaerosols.

## Introduction

Bioaerosols found in working environments such as agricultural facilities [1], sawmills [2], machining plants [3] and dentist clinics [4] can cause respiratory diseases [5]. National Institute for Occupational Safety and Health (NIOSH) reported increased respiratory disease-related mortality in workers exposed to bioaerosols [6]. The exact nature of the harmful components of bioaerosols is still incompletely understood. For example, endotoxins alone cannot explain all the respiratory health effects of exposure to swine confinement buildings [7], [8], [9]. Also, hypersensitivity pneumonitis (HP) such as farmer's lung disease is an allergic response, most frequently to *Saccharopolyspora rectivirgula* (SR), but may require a trigger factor [10]. Thus, the involvement of bioaerosol components in the pathogenesis of respiratory diseases is complex and incompletely understood.

Diseases caused by bioaerosols, including asthma, rhinitis, HP, organic dust toxic syndrome (ODTS) and chronic bronchitis [5], are characterized by distinct immune reactions. While asthma is often characterized by infiltration of eosinophils and neutrophils, workers suffering from hypersensitivity pneumonitis have massive accumulation of lymphocytes in the airways, various degrees of interstitial infiltrates and granuloma formation [11], [12]. Also, ODTS and chronic bronchitis are characterized by the accumulation of neutrophils in the lungs [13]. Thus, characterization of airborne dust components and study of their immunogenic effects is required to further define the causality of bioaerosols in different pulmonary diseases.

We recently documented a high density of archaea (10^8^ per cubic meter of air) in swine confinement buildings, while the airborne bacterial density in that environment was 10^9^ per cubic meter of air [14]. *Archaea* is one of the three domains of living organisms with *Eukarya* and *Bacteria*. Archaeal morphology and gene size are similar to those of bacteria, though their gene information processing resemble that of eukaryotes [15], [16], [17]. Archaea have exclusive membrane lipids, mainly composed of archaeol and caldarchaeol [18], [19], which depending on the species, differentially modulate host's immune responses [20]. Also, archaeal heat-shock proteins can induce immune responses [21], [22]. Yet, immunogenic properties of complete archaea cells, as found in bioaerosols, are unknown. Because archaea are potentially immunogenic and present at high levels in bioaerosols, they could modulate pulmonary immune responses and participate to the pathogenesis of exposure-related airway diseases. Considering the high concentration of airborne archaea in swine confinement buildings, could they be a missing link to explain the diversity or the nature of respiratory immune responses to that environment? Before answering this question, immunological effects of archaea need to be documented.

To study the immunomodulatory effects of the two major archaeal species found in agricultural facilities, i.e. *Methanobrevibacter smithii* (MBS) and *Methanosphaera stadtmanae* (MSS) [14], [23], [24], we mimicked chronic airway exposure to archaea in mice. We developed a model based on an extensively validated protocol previously used to study HP in mice [25], [26], [27]. Since components of archaea can be immunogenic, we hypothesized that archaeal species would induce immunopathological responses in mice. Our results confirm the immunogenic potential of archaea and show that different species found at high concentrations in the air of agricultural working environments have distinct immunogenic properties.

## Materials and Methods

### Preparation of archaea

Archaea were obtained from Agriculture Canada (Robert Forster). Pellets were washed with distilled water and lyophilized. Cells were reconstituted with sterile saline, sonicated 1 minute to break cellular clusters and suspensions were aliquoted and stored at −20°C. Fresh suspensions were thawed on each instillation day.

### Animals

Pathogen-free C57Bl/6 female mice (18–20 g) were obtained from Charles River (St-Constant, QC, Canada). Experimental protocols, care and handling procedures were reviewed and approved by the Animal Experimentation Ethic Committee of Laval University (approval # 2010050-1) and by the Canadian Council on Animal Care.

### Airway exposure protocol

**Figure 1:**
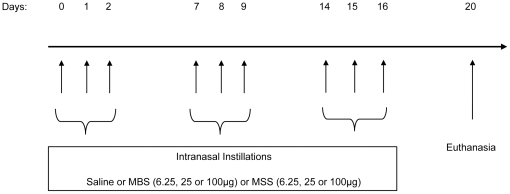
Experimental model. Mice received intranasal instillations of saline or; 6.25 µg, 25 µg, 100 µg of MBS or; 6.25 µg, 25 µg, 100 µg of MSS, once daily, starting on day 0, for 3 consecutive days in a week, during 3 weeks. Mice were euthanized on day 20, 4 days after last archaea instillation.

Mice were anesthetized with isoflurane and instilled intranasally, once daily, on 3 consecutive days per week, for 3 weeks, with 50 µl of saline or with saline containing 6.25, 25 or 100 µg of archaea. Animals were euthanized by ketamine-xylazine overdose 4 days after the last instillation [28] (Figure 1).

### BALF analysis

After cannulation of the trachea, lungs were washed three times with 1 ml of saline solution. Absolute number of immune cells and differential counts were performed as previously described [27], [28] and the frequencies of T cells, B cells and dendritic cells were investigated by flow cytometry. Cells were stained with fluorochrome-labeled antibodies against murine CD4, CD8, CD19, CD11b, CD11c or I-A/I-E (Biolegend, eBiosciences and BD Pharmingen). Fluorescence and autofluorescence were acquired using a FACS Diva-driven FACS Aria (Becton Dickinson) and analyzed with the Flowjo software (Tree star inc.) [29]. The total number of cells was multiplied by frequencies obtained by flow cytometry to compute the absolute number of specific cell subsets.

### Histopathological studies

After bronchoalveolar lavages were performed, lungs were fixed in 10% neutral buffered formalin. The left caudal lobes were embedded in paraffin, sectioned and stained with hematoxylin and eosin as previously described [29]. Blinded histopathological evaluation was performed by a veterinary pathologist (C.T.). Severity grades were given for every histological change with scores ranging from 0 to 5 (0 = null; 1 = minimal, 2 = mild, 3 = moderate, 4 = marked and 5 = severe). Histopathological evaluation was performed on six mice per group and median values were given for every group.

### Immunoglobulin titration

Indirect ELISA was used to measure MBS and MSS-specific IgG levels. Plates were coated with either MBS or MSS cellular suspensions (50 µg lyophilized cells/ml) [30]. After incubating with 100 to 7 812 500 fold plasma dilutions, plates were washed and overlaid with horseradish peroxidase-coupled goat anti-mouse IgG (H+L, Human adsorbed) for chromogenic reaction and quantification of optical density (OD) [30]. Each plate contained blank and internal controls. OD from saline-treated mice was subtracted from OD of experimental samples and curve calculator program (XLfit) was used to determine curves' equations. IgG titers were obtained by calculating plasma titration when (OD sample – OD saline) curves crossed 0. Titers are expressed as −[Log_10_ (serum dilution)].

### Statistical analysis

Data were expressed using mean ± standard error of the mean, or using median with interquartile range for continuous variable. For continuous data, one-way ANOVA was used to compare groups. For some variables, values were log transformed to stabilize variances. In situations where the normality assumptions were unjustified after log transformation, the alternative procedure used was the rank transformation using the ordinary F test from the one-way ANOVA. Reported p-values are based on these transformations. The variance assumptions were verified using the Brown and Forsythe's variation of Levene's test statistic. The univariate normality assumptions were verified with the Shapiro-Wilk tests. Histopathology data were analyzed with one-way ANOVA with a Poisson distribution. Goodness of fit of Poisson distribution was tested using the Chi-Square test. The results were considered significant with p-values ≤0.05. All analyses were conducted using the statistical package SAS v9.1.3 (SAS Institute Inc, Cary, NC, U.S.A.).

## Results

### Histopathological alterations in the lungs differ between archaeal species

**Figure 2:**
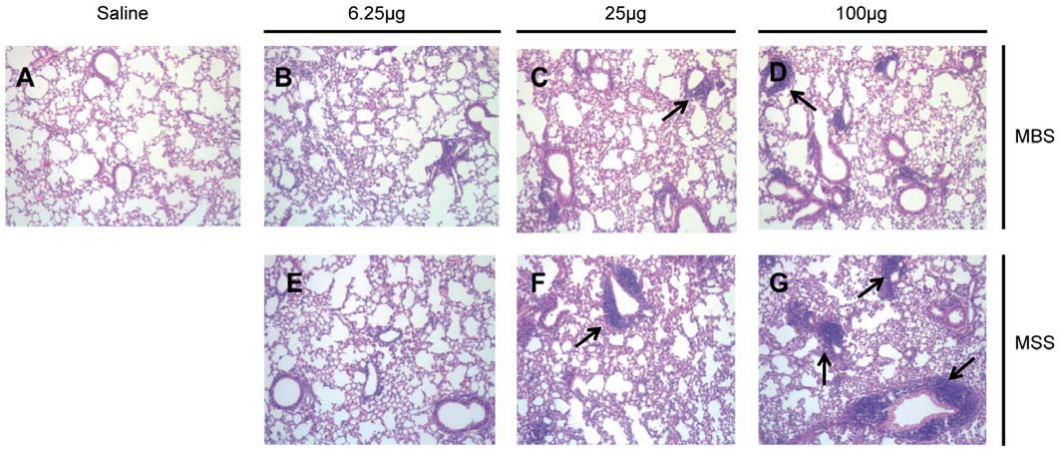
MBS and MSS induce dose-dependent histopathological alterations in the lungs. Hematoxylin and eosin-stained sections of the lungs obtained from A) mice instilled with saline 3 times a week for 3 weeks; mice instilled with B) 6.25, C) 25 or D) 100 µg of MBS 3 times a week for 3 weeks; mice instilled with E) 6.25, F) 25 or G) 100 µg of MSS 3 times a week for 3 weeks. MSS strongly induces the formation of tertiary lymphoid organ-like structures (arrows) compared to MBS. Specimens shown are representative of 6 individual observations per group.

The ability of archaeal species to induce histopathological alterations in our model of airway exposure (Fig. 1) was first evaluated (Fig. 2 and Table 1). While saline-treated mice had no sign of inflammation, MBS and MSS induced dose-dependent histological alterations. At the lowest dose tested (6.25 µg), the inflammatory reaction was seen as multifocal perivascular infiltrates composed of granulocytes with occasional involvement of the vascular wall (vasculitis). At that dose, perivascular granulocyte infiltrates were mainly seen with MSS (median score of 1.5/5). Mid (25 µg) and high (100 µg) archaea doses caused thickening of the alveolar septa by macrophages, mononuclear cells and type II pneumocytes. Thickening of the alveolar septa was always more severe with MSS (scores from 2/5 to 3/5) compared to MBS-treated mice (scores from 0/5 to 1/5). MSS also induced more severe peribronchiolar and/or perivascular mononuclear cellular infiltrate compatible with tertiary lymphoid organ formation (perivascular score: 2/5, 3/5 and 4/5 for the 6.25, 25 and 100 µg MSS doses, respectively). Thus, histological evaluation showed the ability of archaeal species to induce histopathological alterations in lungs with MSS inducing more severe responses than MBS.

**Table 1:** Histopathological alterations of mice lungs exposed to MBS or MSS.

	Saline	MBS			MSS		
Criteria		6.25 µg	25 µg	100 µg	6.25 µg	25 µg	100 µg
Perivascular infiltration of mononuclear cell	0	1	2	3^a^	2	3	4^a^
Perivascular infiltration of granulocytes	0	0	0	0	1.5	0	0
Peribronchial infiltration of mononuclear cell	0	0^a^	1^a^	0^a^	1^b^	1^b^	1^b^
Thickening of alveolar septa	0	1^a^	1^a^	2^a^	2^b^	2^b^	3^b^
Macrophage accumulation	0	0.5	1.5	2^a^	1.5	1	2^a^

Results are expressed as median scores for each group, graded on a scale from 0 (no alteration) to 5 (severe alterations). Letters represent statistical differences between each dose of MBS and MSS within a same criteria. Six mice per group were analyzed. p<0.05.

### Nature and numbers of leukocytes found in BALF differ between archaeal species

**Figure 3:**
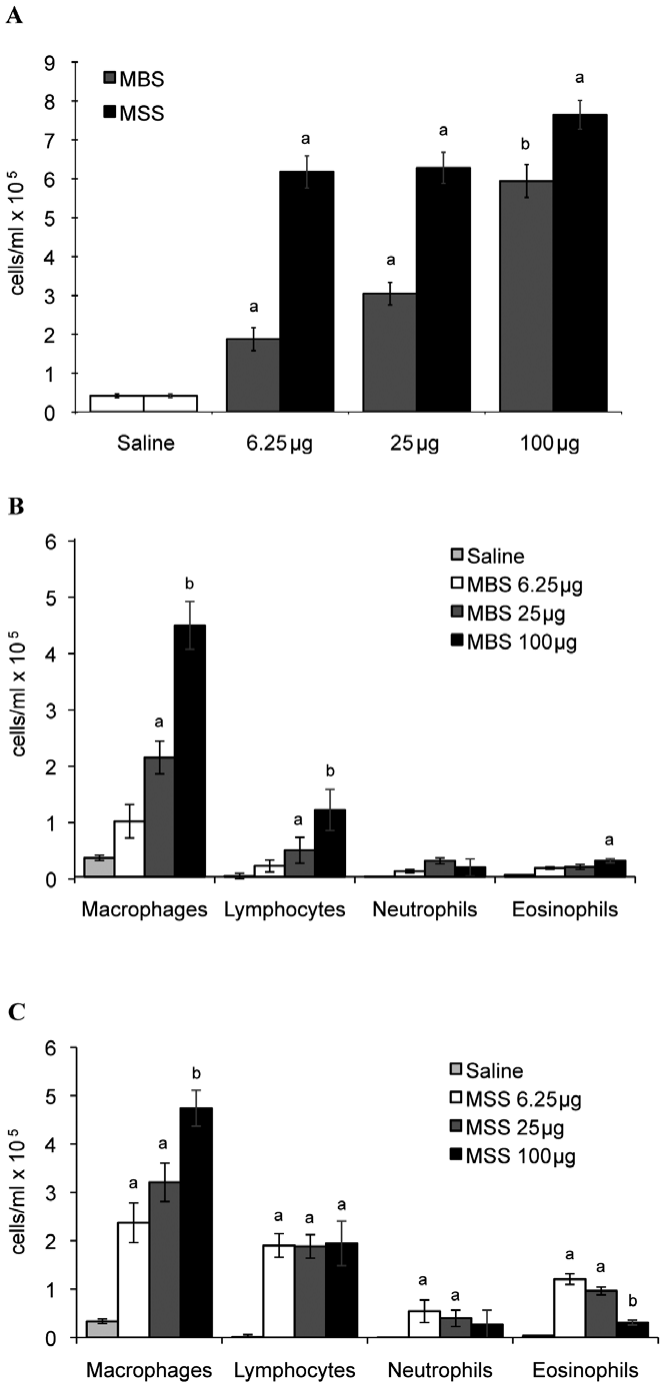
Quantification of leukocyte subsets in the BALF of mice instilled with increasing doses of two archaeal species. A) Total immune cell numbers in BALF of mice instilled with 6.25, 25 or 100 µg of MBS or MSS for 3 weeks. B) Leukocyte subset numbers in BALF of mice exposed to 6.25, 25 and 100 µg of MBS for 3 weeks. C) Leukocyte subset numbers in BALF of mice exposed to 6.25, 25 and 100 µg of MSS for 3 weeks. Results expressed as average ± SEM. For both archaeal species, BALF cell subtypes were mainly composed of macrophages and lymphocytes. MSS also induced a significant accumulation of eosinophils and of neutrophils. Letters a and b represent statistical differences between treatment regimens within a same cell type. Results from two pooled experiments obtained with similar results are presented and 14 to 22 mice per group were analyzed. p<0.05.

In view that histopathological features differed between archaeal species, we determined how MBS or MSS modulated leukocyte populations in the airways (Fig. 3). BALF from control mice contained low cell numbers (0.41×10^5^ cells/ml), which were mainly composed of macrophages (>90%). Instillation of 6.25, 25 and 100 µg of MBS induced increasing accumulation of total cells (Fig. 3A; average values of 1.87×10^5^, 3.04×10^5^, 5.94×10^5^ cells/ml respectively), macrophages (Fig. 3B; average values of 0.98×10^5^, 2.11×10^5^, 4.46×10^5^ cells/ml respectively) and lymphocytes (Fig. 3B; average values of 0.19×10^5^, 0.47×10^5^, 1.18×10^5^ cells/ml respectively) in BALF. BALF from mice exposed to 6.25 µg of MSS (Fig. 3A) showed strong accumulation of leukocytes (average of 6.69×10^5^ cells/ml) in the airways and higher doses induced a similar cell response to the 6.25 µg group. High numbers of BALF macrophages/monocytes (average of 3.44×10^5^ cells/ml) and lymphocytes (average of 1.91×10^5^ cells/ml) were observed at every dose of MSS (Fig. 3C). MSS also induced a negative dose-response accumulation of eosinophils (average of 1.21×10^5^ cells/ml with the 6.25 µg dose to 0.31×10^5^ cells/ml with the 100 µg dose), and neutrophils (average of 0.54×10^5^ to 0.27×10^5^ cells/ml). The intensity and the nature of the leukocyte responses thus differed between the two archaeal species.

### Archaea induce an antigen-specific adaptive response

**Figure 4:**
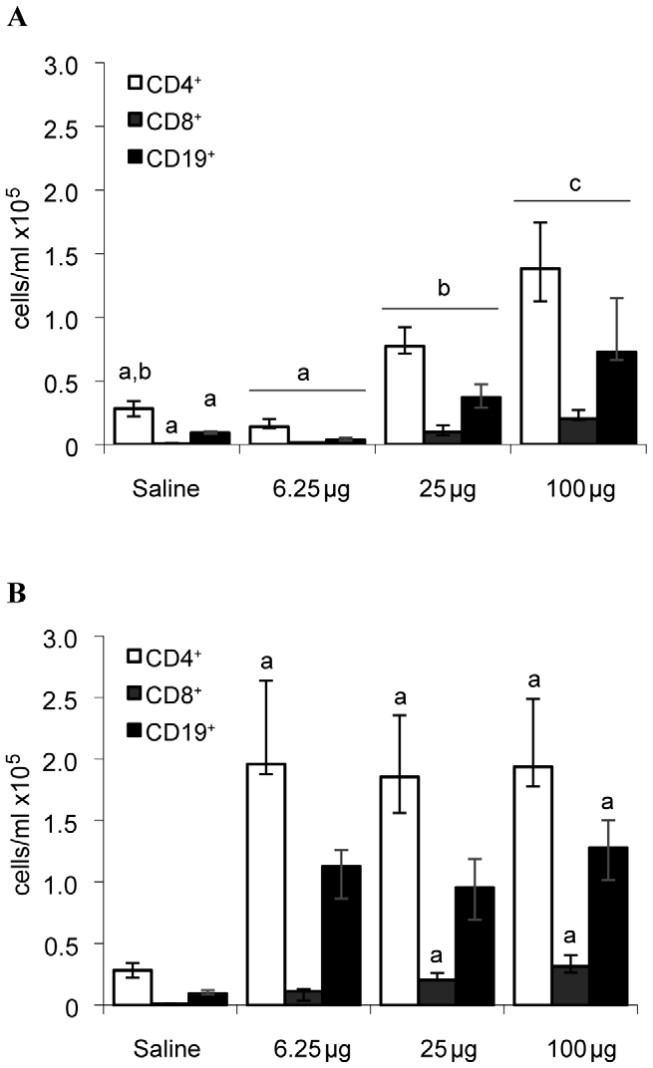
Dominance of CD4^+^ T lymphocytes and CD19^+^ B lymphocytes in BALF of mice instilled with archaeal species. The numbers of CD4^+^ T cells, CD8^+^ T cells and CD19^+^ B cells were analyzed in BALF of mice exposed for 3 weeks to 6.25, 25 and 100 µg of A) MBS or B) MSS. Compared to MBS, MSS induced strong CD4^+^ helper T cell and CD19^+^ B cell responses in the airways, which plateaued from the lowest dose instilled. Results are expressed as median ± interquartile intervals. Letters a and b represent statistical differences between treatment regimens within a same cell type. Six mice per group were analyzed. p<0.05.

Lymphocytes were a major cell type found in BALF in response to archaeal species. We thus investigated how adaptive immunity was modulated. BALF from mice instilled with saline contained low levels of lymphocytes (0.49×10^5^ cells/ml) (Fig. 3B–C). MBS induced a dose-dependent accumulation of lymphocytes in BALF (Fig. 3B), which was characterized by high median numbers of CD4^+^ helper T cells (Fig. 4A) i.e. 0.14×10^5^, 0.77×10^5^ and 1.38×10^5^ cells/ml for the 6.25, 25 and 100 µg doses, respectively. B cells (Fig. 4A) were the second most prevalent lymphocyte subset with median values of 0.04×10^5^, 0.37×10^5^ and 0.73×10^5^ cells/ml for the 6.25, 25 and 100 µg doses, respectively. On the other hand, CD8^+^ T cells were found at low numbers with maximal median accumulation of 0.20×10^5^ cells/ml in the 100 µg MBS-treated group. BALF of mice instilled with MSS (Fig. 4B) also had high median numbers of CD4^+^ lymphocytes (1.85×10^5^ to 1.96×10^5^ cells/ml) and B lymphocytes (0.96×10^5^ to 1.28×10^5^ cells/ml) for the three MSS doses studied. Similar to MBS, MSS induced a modest cytotoxic response with medians of 0.11×10^5^, 0.20×10^5^ and 0.31×10^5^ CD8^+^ T cells for the 6.25, 25 and 100 µg doses, respectively.

**Figure 5:**
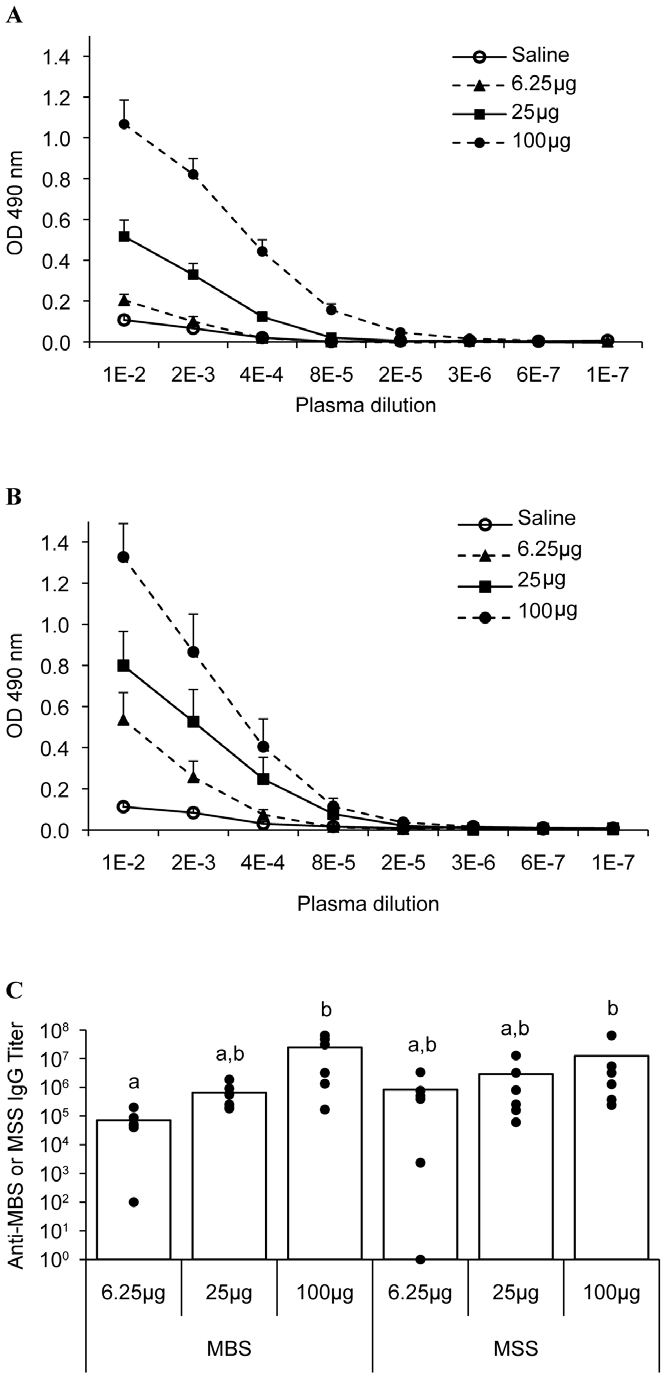
MBS and MSS induce a dose-dependent production of antigen-specific IgG in plasma. Titers of antigen-specific IgG were measured by ELISA in plasma of mice exposed to 6.25, 25 and 100 µg of A) MBS or B) MSS. C) Titers are expressed as the logarithmic inverse of plasma dilutions. Both archaea induced a dose-responsive generation of archaea-specific antibodies. Results are expressed as mean ± SEM. Letters a and b represent statistical differences between treatment regimens. Six mice per group were analyzed. p<0.05.

Since both archaeal species induced a strong B cell response, antigen-specific IgGs were titrated in plasma (Fig. 5). Low immunoreactivity was detected in the saline-treated groups (Fig. 5A–B) and direct dose-relationships were observed between increasing doses of MBS or MSS and antigen-specific IgG titers (Fig. 5A–C). Although leukocyte subsets and numbers differed between MBS and MSS groups (Figs. 3 and 4), both archaeal species lead to similar levels of antigen-specific IgG (Fig. 5C). Thus, both archaea induced a full immune response.

### MSS triggers higher accumulation of myeloid dendritic cells than MBS

**Figure 6:**
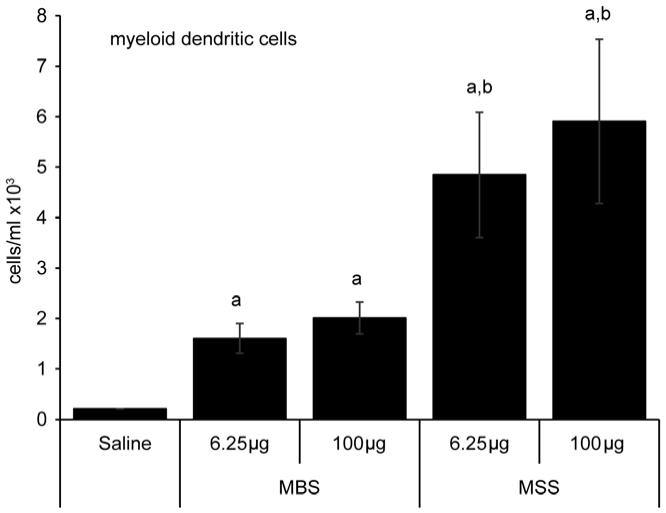
MSS induces stronger myeloid dendritic cells response than MBS in the airways of mice. Flow cytometric analyses were performed on BALF cells of mice exposed to 6.25 or 100 µg of MBS or MSS for 3 weeks. Compared to saline-treated mice, MBS did not induce significant accumulation of myeloid dendritic cells in the airways. Myeloid dendritic cell response was increased with MSS. Results are expressed as absolute numbers of cells (average ± SEM). Six mice per group were analyzed. a: p<0.05, represent statistically differences compared to saline. b: p = 0.08.

Because MSS appeared to be more immunogenic than MBS, differences were confirmed by quantifying the myeloid dendritic cell response. Myeloid dendritic cells were defined as CD11c^+^ autofluorescence^low^, CD11b^+^ MHCII^hi^. BALF from saline-treated mice had very low levels of myeloid dendritic cells (average of 0.21×10^3^ cells/ml) (Fig. 6). Similar to saline-treated mice, BALF from mice submitted to the 6.25 and 100 µg MBS instillation protocols contained low average numbers of myeloid dendritic cells/ml i.e. 1.6×10^3^ and 2.0×10^3^ respectively. MSS led to a higher myeloid dendritic cell response for every dose studied (4.8×10^3^ and 5.9×10^3^ cells/ml) when compared to MBS (Fig. 6). Thus, immunogenicity of archaeal species correlates with their ability to induce a strong myeloid dendritic cell response.

## Discussion

Respiratory diseases caused by bioaerosols include infections, toxic, and hypersensitive reactions. Archaea are a major component of bioaerosols in agricultural environments [14], but environmental exposure of human to archaea and their impact on respiratory diseases remain undefined. This study shows that both MBS and MSS are immunogenic and trigger a full immune response leading to the generation of antigen-specific antibodies. Importantly, we determined that the nature and intensity of the immune responses differed between archaeal species, with MSS inducing a more severe response than MBS at all doses tested. Most interestingly, MSS but not MBS induced a granulocytic response and that response inversely correlated with the size of the inoculum. Potent immunogenicity of MSS was confirmed by a strong accumulation of myeloid dendritic cells in the airways compared to MBS. Our results are thus the firsts to support archaeal species as potential etiological agents in exposure-related pulmonary diseases.

Archaeal species commonly found in bioaerosols are immunogenic. Archaeal species used in the study are methanogens, extremely sensitive to oxygen, and therefore non-viable and non-infectious when found in bioaerosols. *Archaea* have unusual membrane lipids [18], [31], [32], [33] compared to *Bacteria* and *Eukarya*, which are potent adjuvant molecules for humoral, mucosal and systemic immune responses [20], [31], [33], [34]. In the context of testing new adjuvants, Krishnan et al. showed that intraperitoneal injection of archaeosomes (liposomes made of archaeal lipids) induced the recruitment and activation of macrophages and dendritic cells in mice [35]. This is in agreement with our current results obtained with total archaea cells. Indeed, we show that intranasal delivery of MBS and MSS species induced the accumulation of dendritic cells and expansion of lymphocytes in pulmonary draining mediastinal lymph nodes (Data not shown, Marsolais and Blais Lecours) and accumulation of tertiary lymphoid organ-like structures in the lungs (Fig. 2). Patel et al. [20] showed that intranasal administration of ovalbumin encapsulated in MBS archaeosomes elicited a strong systemic immune response in a mouse model, and Yamabe et al. showed that archaea can induce a specific IgG response in humans [36]. In agreement with these results, we show that multiple lymphocyte expansion sites lead to a pronounced accumulation of helper T cells and B cells in the airways and to an antigen-specific IgG response. This is consistent with archaea-specific IgGs in the sera of humans exposed to the air of agricultural buildings contaminated with archaeal species (Blais Lecours, data not shown).

Immunopathological features observed in our model resemble those of hypersensitivity pneumonitis (HP) and of murine models of HP [37]. Indeed, HP is a chronic disease caused by inhaled agents and is characterized by strong CD4^+^ T lymphocyte accumulation in the BALF and noncaseating granulomas in the lung tissues. Patients suffering from HP also have high serum antigen-specific IgG titers. Intriguingly, one archaeal species (MSS) also led to eosinophilic inflammation. In view that eosinophilic inflammation is usually associated with the development of asthma and that archaeal species induce a strong T cell response, it is tempting to speculate that archaeal species might be linked to the development of exposure-related airway diseases including HP and occupational asthma. Moreover, our study reveals that not only the nature, but also the dose of the inhaled agent could determine the nature of an exposure-related respiratory disease, with increasing doses of MSS leading to a downregulation of eosinophil accumulation in the airways. Mechanisms of eosinophilic inflammation downregulation by increasing doses of MSS are currently being investigated.

Our observation that MSS has a stronger immunogenic potential than MBS does not reconcile with results obtained with purified archaeal lipids [33]. Indeed, MSS archaeosomes induce a mild humoral response, relatively to MBS-derived archaeosomes, by mechanisms relying on uptake efficiency of macrophages, but that remains controversial [38], [39]. Discrepancies between the current study and experiments performed with purified lipids are likely explained by non-lipid immunogenic components of archaea, including proteins [21], [22]. Thus, immunogenicity of complete archaea cells cannot be explained only by the immunomodulatory properties of their lipids.

Archaeal species differentially modulate dendritic cells in the airways. Dendritic cells are potent antigen presenting cells that initiate the immune response [40], [41]. Myeloid dendritic cells quantitatively modulate the pulmonary immunity, with high numbers of myeloid dendritic cells often associated with a strong immune response [29], [42]. Our results show that MSS, but not MBS, strongly stimulate myeloid dendritic cell accumulation in the airways, possibly explaining the strong immunogenic properties of MSS. Thus, different archaeal species possess different immunogenic properties that are linked to their ability to induce or sustain a myeloid dendritic cell response in the airways.

### Conclusion

We show that two archaeal species recently found in the bioaerosols of several working environments induce diverse histopathological alterations in a murine model of airway exposure. When administered in the airways, archaeal species induce a full immune response with the generation of antigen-specific antibodies. MSS caused significant airway accumulation of eosinophils and neutrophils, which inversely correlated with the size of the inoculum. Importantly, airways of MBS-instilled mice were devoid of granulocytes, revealing that an interaction between the archaeal species and the quantity of archaea inoculated determines the type of airway inflammation. Moreover, different immunogenic properties of archaeal species were confirmed by their differential ability to induce myeloid dendritic cell accumulation in the airways. This is the first study documenting the immunomodulatory effects of archaea, which were only recently discovered as prominent bioaerosol components. Our findings suggest that archaeal species found in bioaerosols could contribute to the diversity of exposure-related airway diseases. Further studies are required to define this contribution.
